# Supplementary material for: Voltammetric analysis for distinguishing portal hypertension-related from malignancy-related ascites: A proof of concept study
Source: PLoS One. 2020 May 21;15(5):e0233350. doi: 10.1371/journal.pone.0233350 (PMC7241828; doi:10.1371/journal.pone.0233350)
Supplement: Attachment — Submitted filename: Response to reviewers.docx [file pone.0233350.s002.docx]

Dear Editor,

we thank you for the opportunity to revise our paper entitled “Voltammetric analysis for distinguishing portal hypertension-related from malignancy-related ascites: a proof of concept study”. The suggestions offered by the reviewers have been very helpful, and the paper has been revised accordingly.

We hope you will find our manuscript improved and finally suitable for publication in the Journal.

Best regards,

Antonio De Vincentis

**Answer to the comments**

**Reviewer #1:** I read with interest this manuscript. The study by Antonio De Vincentis described Voltammetric analysis for distinguishing portal hypertension-related from malignancy-related ascites. The manuscript is well written and presented, conclusions are sound. The topic is of interest to the scientific community. I have only one minor revision: -The authors should discussion in the conclusion how they intend to further improve the accuracy of this technique in future studies. This is a proof of concept study and I think that is important to give future prospects for studies.

**Answer to the comment:** We are glad the reviewer appreciated our work and we thank him/her for the comment. The analysis of electrochemical properties of a given fluid may account upon a huge variety of experimental conditions, i.e., broadly speaking, of experimental electrical fields. [26] Thus, comparing different experimental conditions to identify that or those yielding the best discriminatory capacity is expected to further simplify the experimental procedure as well as to increase its diagnostic accuracy. Furthermore, a more detailed characterization of ascites will allow verify whether a given e-tongue finding, i.e., a well-defined electrochemical pattern, recognizes selected determinants and, thus, points at a distinctive chemical composition. Finally, portal hypertension is a heterogeneous condition as for etiology and severity, and neoplastic ascites also has many determinants and variable physicochemical properties. Thus, expanding the library to compare different and well-sized categories of either hemodynamic or neoplastic ascites is expected to provide potential insight into the diagnostic properties of the e-tongue. Indeed, we cannot exclude that selected clinical and pathophysiological conditions will benefit the most from the classificatory and prognostic properties of the e-tongue. In this perspective, the follow up of patients might be of interest to test the prognostic properties of the voltammetric findings versus outcomes such as responsivity of ascites to diuretic therapy or survival. These considerations are now available in the “Discussion” section on pages 13 to 14, lines 290 to 300, and in the “Conclusion” section on page 14, lines 319 to 321.

**Reviewer #2:** The paper its related to a study, through Voltammetric analysis, for distinguishing portal hypertension-related from malignancy related ascites. The paper is interesting, but authors should convince the reviewer that the proposed method is valid, by comparing it with the literature. Moreover, better information on experimental set-up (BioNote), in particular, technical data and some figures related to measurements, must be given for a broader audit of the paper (sensor researchers).

**Answer to the comment:** We thank the reviewer for his/her comment. Concerning the comparison of BIONOTE with scientific literature on the field, it is worth remarking that no experiment using this sensor in ascitic fluid has been reported so far. However, we had previously tested BIONOTE for classification of other biological fluid. [18-20] In the revised manuscript, the measurement process using BIONOTE has been illustrated in a figure, i.e., Figure 1) to make it clearer to any kind of reader. Generally, chemical sensors are based on specific or non-specific sensing materials. The kind of sensing material could contribute to obtain selective or non-selective sensors. Accordingly, Biosensors are specific sensors and their sensing mechanisms is so called “key-lock”. Using a sensing material on the transducer requires particular processes of manufacturing in order to obtain reproducible and repeatable sensors. In our case we used a non-functionalized sensor, in particular no sensing materials are used on the electrode surface.

The sensing material has an advantage for bio-medical applications (in general), but two disadvantages for the specific application here presented. The advantage consists in the possibility of selecting a well defined sensing element interacting with a specific biomarker or a specific compound of interest for the sample under test, increasing sensor performance in terms of sensitivity, resolution and, obviously, selectivity. However, the approach of this paper is the fingerprinting and not the identification and/or quantification of specific biomarkers. Moreover, the process of covering the surface of the sensor with a chemical interactive material requests another technological step which affects the overall reproducibility of the sensor.

The commercial electrode (Au, Pt, Ag) with a dedicated electronic interface developed by the Unit of Electronics for Sensor Systems of Campus Bio-Medico di Roma guarantees the reproducibility and repeatability of measurement as shown in other papers [25,26,35]. In the future this kind of approach could reduce the operational costs of the device. We did not include these comments in the text because they seems more suited for a pure technological journal. However, the bases of the voltammetric analyses are reported in detail in the above cited references and in the following three.

**References**

18. Lelli D, Pedone C, Alemanno P, Bertini A, Di Gioia C, Fazzina S, et al. Voltammetric analysis for fast and inexpensive diagnosis of urinary tract infection: a diagnostic study. J Transl Med. 2018;16:17.

19. Lelli D, Pedone C, Frezzotti E, Pennazza G, Santonico M, Grasso S, et al. Use of voltammetric analysis for fast and objective discrimination of the etiology, evolution, and bacterial infection of lower limb ulcers. Wound Repair Regen Off Publ Wound Heal Soc Eur Tissue Repair Soc. 2019;27:288–91.

20. Santonico M, Frezzotti E, Incalzi RA, Pedone C, Lelli D, Zompanti A, et al. Non-invasive monitoring of lower-limb ulcers via exudate fingerprinting using BIONOTE. Sens Actuators B Chem. 2016;C:68–74.

25. Pennazza G, Santonico M, Vollero L, Zompanti A, Sabatini A, Kumar N, et al. Advances in the Electronics for Cyclic Voltammetry: the Case of Gas Detection by Using Microfabricated Electrodes. Front Chem [Internet]. 2018 [cited 2019 Aug 30];6. Available from: <https://www.ncbi.nlm.nih.gov/pmc/articles/PMC6095978/>

26. Santonico M, Pennazza G, Grasso S, D’Amico A, Bizzarri M. Design and test of a biosensor-based multisensorial system: a proof of concept study. Sensors. 2013;13:16625–40.

35. Pennazza G, Santonico M, Zompanti A, Parente FR, Ferri G, D’Amico A. Design and Development of an Electronic Interface for Gas Detection and Exhaled Breath Analysis in Liquids. IEEE Sens J. 2018;18:31–6.
